# Impact of cirrhosis on long‐term survival outcomes of patients with intrahepatic cholangiocarcinoma

**DOI:** 10.1002/cam4.4740

**Published:** 2022-04-12

**Authors:** Jian Wang, Yiwen Qiu, Yi Yang, Shu Shen, Ming Zhi, Bo Zhang, Wentao Wang

**Affiliations:** ^1^ Department of Liver Surgery & Liver Transplantation Center, West China Hospital Sichuan University Chengdu China; ^2^ Department of Hepatobiliary Surgery II The People's Hospital of Ganzi Tibetan Autonomous Prefecture Kangding China; ^3^ Department of Gastrointestinal Surgery, West China Hospital Sichuan University Chengdu China; ^4^ Sanya People's Hospital/West China Sanya Hospital Sanya China

**Keywords:** cirrhosis, intrahepatic cholangiocarcinoma (ICC), prognosis, propensity score matching (PSM), SEER database

## Abstract

**Background:**

The correlation between cirrhosis and the long‐term oncological outcome in intrahepatic cholangiocarcinoma (ICC) is debatable, and this study aimed to explore the impact of cirrhosis on the long‐term prognosis of patients with ICC.

**Methods:**

A total of 398 ICC patients were identified in the Surveillance, Epidemiology, and End Results (SEER) database from 2000 to 2018. The diagnosis of cirrhosis was based on the Ishak fibrosis score provided by the SEER database. Propensity score matching (PSM) and inverse probability of treatment weighting (IPTW) analysis were performed to minimize the potential confounders. Overall survival (OS) and cancer‐specific survival (CSS) were observed, and the Cox regression model was used to select potential factors that affect the prognosis of the patients with ICC.

**Results:**

Of the included patients, there were 142 patients and 256 patients in the cirrhotic and noncirrhotic groups, respectively. Additionally, 299 of 398 patients (75.1%) died following a median follow‐up of 19 months (interquartile range [IQR], 7, 43). The OS and CSS indicated advantage trend in the noncirrhotic group than the cirrhotic group in either the original cohort (OS: 17 vs 12 months, *p* = 0.023; CSS: 26 vs 15 months, *p* = 0.004) or the PSM (OS: 17 vs 12 months, *p* = 0.52; CSS: 22 vs 14 months, *p* = 0.15) or IPTW (OS: 20 vs 13 months, *p* = 0.163; CSS: 22 vs 15 months, *p* = 0.059) cohorts. Subgroup analyses displayed that the prognosis of patients who experienced surgery for ICC in the noncirrhotic group was better than that of the cirrhotic group with regard to OS and CSS.

**Conclusions:**

Collectively, it seems that the noncirrhotic patients have similar relative OS but better CSS compared with that of the cirrhotic patients.

## Graphical abstract

In this study, we applied the statistical method of PSM to reduce the risk of an inherent selection bias based on the patients with intrahepatic cholangiocarcinoma (iCCA) extracted from the Surveillance, Epidemiology, and End Results (SEER) database for exploring the impact of cirrhosis on the prognosis of iCCA.

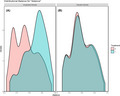

## 1 INTRODUCTION

Intrahepatic cholangiocarcinoma (ICC) is a kind of hepatobiliary tumor derived from the intrahepatic bile ducts that account for 10%–15% of all primary hepatic malignant tumors and approximately 20% of cholangiocarcinoma/gallbladder cancers. ICC is characterized by highly aggressive invasiveness, few treatment options, and a dismal prognosis. Complete surgical resection with negative margins (R0) is still the main effective management for patients with ICC, with a 5‐year survival rate after curative‐intent resection of ICC still unsatisfactory. In the past few decades, the incidence and related mortality of ICC have been reported to be dramatically increasing worldwide.^1^, ^2^, ^3^

Existing evidence has found that cirrhosis is one of the strong risk factors for ICC, and a large proportion of patients with ICC have cirrhosis caused by hepatitis B (HBC), hepatitis C (HCV), steatohepatitis, etc. However, noncirrhotic factor was identified in most patients with ICC.^4^, ^5^, ^6^ Currently, cirrhosis is viewed as an adverse short‐ and long‐term prognostic factor for ICC patients in several studies.^7^, ^8^, ^9^, ^10^, ^11^, ^12^, ^13^, ^14^, ^15^, ^16^, ^17^ It has been well defined that cirrhosis increases a high incidence of complications such as prolonged hospital length of stay and liver failure following hepatectomy, which results in reducing the opportunity to perform surgical resections for ICC patients with cirrhosis.^7^, ^8^, ^9^, ^10^ In addition, several studies have reported a correlation between cirrhosis and worse oncological outcomes of ICC.^11^, ^12^, ^13^, ^14^, ^15^, ^16^, ^17^ However, several other studies have reported that cirrhosis itself does not pose an unfavorable effect on the prognosis of ICC patients.^18^, ^19^ Collectively, whether cirrhosis hurts the oncological results of patients with ICC is still ambiguous and controversial.

Therefore, based on the above‐mentioned research status, the objective of this study was to investigate the impact of cirrhosis on the prognosis of ICC patients identified from the Surveillance, Epidemiology, and End Results (SEER) database.

## 2 METHODS

### 2.1 Patient selection

Data on ICC patients with concurrent cirrhosis were obtained from the SEER database, Public Use Data, for the period 2000–2018. All patients in the SEER‐18 registries during 2000–2018, which covers approximately 28% of the US population, were potentially eligible for inclusion in our study. Due to SEER research data being publicly accessible, this study does not need to require informed patient consent. We used data submitted in May 2021 from the SEER registry through SEER*Stat software (version 8.3.9, http://seer.cancer.gov/seerstat/).

**FIGURE 1:**
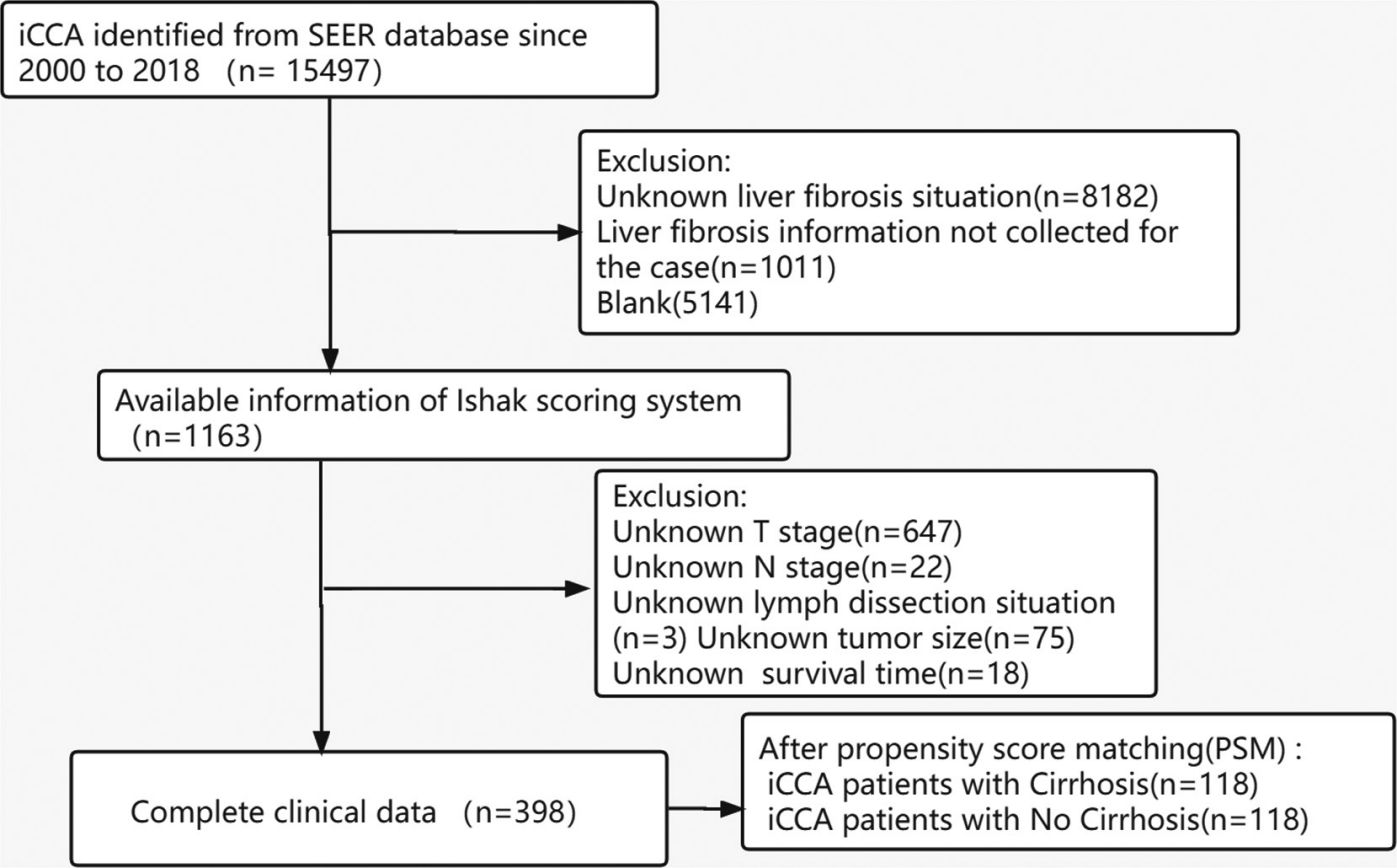
The flow chart of the selection process for the study cohort

The primary cancer site was restricted to the primary site code for the liver (C22.0) and intrahepatic bile duct (C22.1) according to the International Classification of Disease for Oncology, 3rd Edition (ICD‐O‐3), along with the histological code for cholangiocarcinoma (8160, 8161,8180). As shown in the flow diagram in Figure 1, we finally included patients pathologically diagnosed from 2010 to 2018 with available Fibrosis Score Recode (2010^+^), demographics, 7th edition of the AJCC TNM staging system, tumor information, and treatment information. We defined the Ishak 5–6 stage as cirrhosis, whereas the Ishak 0–4 stage was defined as no cirrhosis.^20^

### 2.2 Statistical analysis

The comparisons of continuous and categorical variables were the ANOVA and the chi‐squared tests, respectively. Survival curves were calculated using the Kaplan–Meier method and compared via the log‐rank test.

The variables used in PSM analysis were based on sex, race, the 7th edition of the AJCC staging system, T stage, N stage, M stage, surgery, lymph dissection, tumor size, marital status, radiation, chemotherapy, and age. OS and CSS were compared between the two groups in the PSM population by a log‐rank test, while a caliper width of 0.01 was used to perform one‐to‐one matching between the groups.^21^ Consistent with PSM, IPTW‐adjusted analysis was used to minimize the potential confounders. In the IPTW method, weights are assigned to patients, creating pseudo‐populations where cirrhosis is independent of covariates. The entire statistical analysis design in this research has been described in the previous study.^22^

All analyses were performed using RStudio (https://www.rstudio.com/products/rstudio/download/) in the context of R version 4.0.3 (https://www.r‐project.org/). The value of *p* < 0.05 in a two‐tailed test was considered statistically significant.

## 3 RESULTS

**FIGURE 2:**
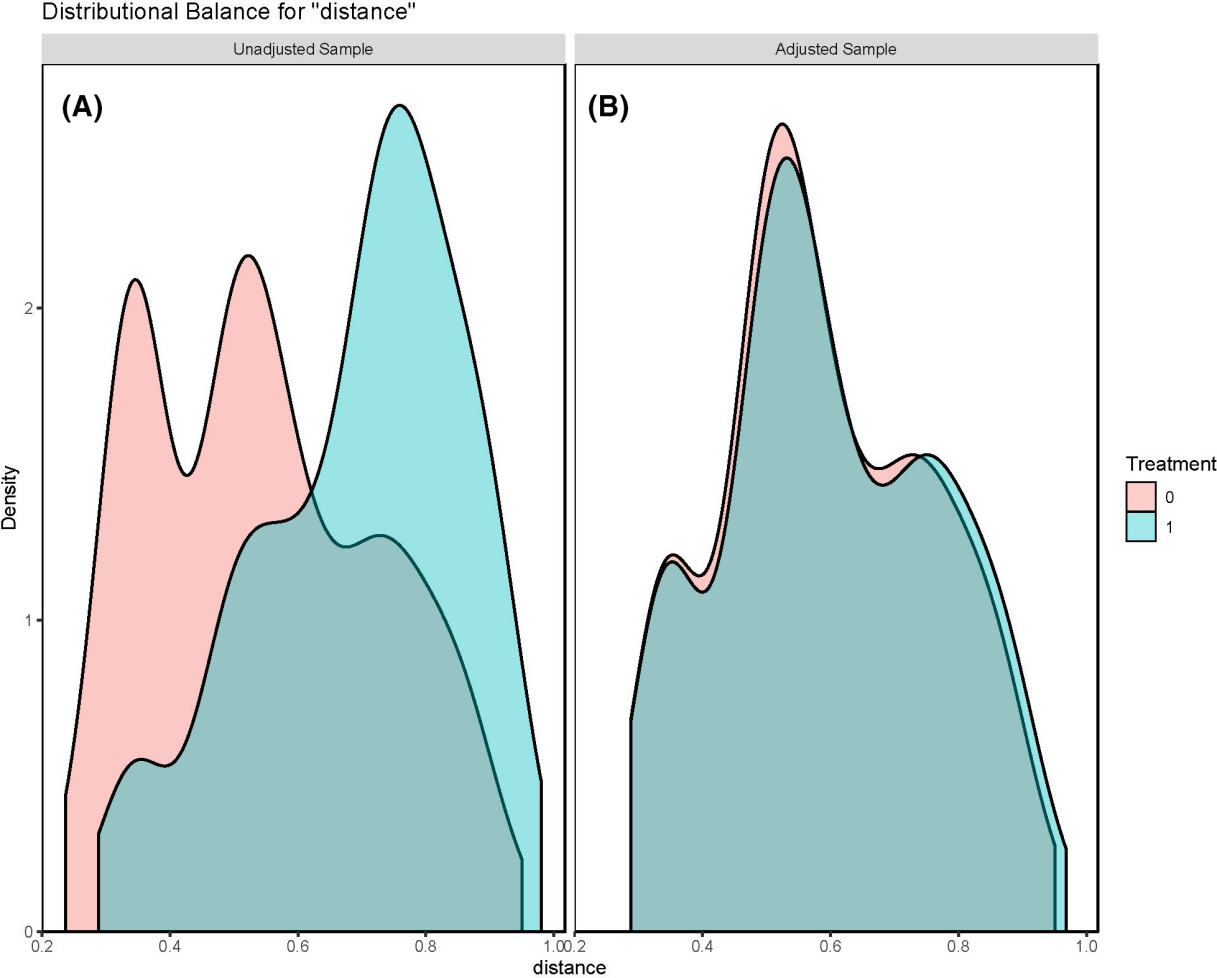
Density function shows the well‐balance distribution for baseline characteristics before (A) and following (B) propensity score matching (PSM). And more overlapping parts indicate more well‐balanced clinicopathological characteristics between the noncirrhotic and cirrhotic groups

A total of 398 patients with ICC identified from the SEER database were included in this retrospective study. The demographic features and clinicopathological characteristics of these ICC patients included in the study are summarized in Table 1. Before matching, the cirrhotic and noncirrhotic groups were 142 (35.7%) and 256 (64.3%), respectively, while 194 patients (48.7%) received surgery, and 204 patients (51.3%) received no surgery. Collectively, the ICC patients with cirrhosis were characterized by younger, earlier AJCC TNM staging, male predominance, a smaller number of nodes dissection intraoperatively, and a higher proportion of nonsurgical patients than that of the noncirrhotic group. As shown in Figure 2, the PSM‐adjusted population displayed similar but more well‐balanced baseline clinicopathological characteristics in the groups of noncirrhotic and cirrhotic patients with ICC compared with that in the original population.

**TABLE 1:** Baseline characteristics of patients with ICC in the whole cohort and the matched cohort

Variables	Levels	Overall (*n* = 398)	Unmatched		*p*	PSM		*p*
			No cirrhosis (*n* = 256)	Cirrhosis (*n* = 142)		No cirrhosis (*n* = 118)	Cirrhosis (*n* = 118)	
Sex (*n*, %)	Female	171 (43.0)	129 (50.4)	42 (29.6)	<0.001	41 (34.7)	42 (35.6)	1
	Male	227 (57.0)	127 (49.6)	100 (70.4)		77 (65.3)	76 (64.4)	
Race (*n*, %)	Black	20 (5.0)	12 (4.7)	8 (5.6)	0.02	5 (4.2)	8 (6.8)	0.468
	Other	64 (16.1)	51 (19.9)	13 (9.2)		18 (15.3)	13 (11.0)	
	White	314 (78.9)	193 (75.4)	121 (85.2)		95 (80.5)	97 (82.2)	
Grade (*n*, %)	III/IV	94 (23.6)	64 (25.0)	30 (21.1)	0.063	30 (25.4)	28 (23.7)	0.226
	I/II	155 (38.9)	107 (41.8)	48 (33.8)		31 (26.3)	43 (36.4)	
	Unknown	149 (37.4)	85 (33.2)	64 (45.1)		57 (48.3)	47 (39.8)	
7th AJCC TNM (*n*, %)	I	136 (34.2)	85 (33.2)	51 (35.9)	0.031	48 (40.7)	41 (34.7)	0.443
	II	88 (22.1)	53 (20.7)	35 (24.6)		29 (24.6)	27 (22.9)	
	III	21 (5.3)	16 (6.2)	5 (3.5)		6 (5.1)	5 (4.2)	
	IVA	64 (16.1)	51 (19.9)	13 (9.2)		5 (4.2)	12 (10.2)	
	IVB	89 (22.4)	51 (19.9)	38 (26.8)		30 (25.4)	33 (28.0)	
T stage (*n*, %)	T1	160 (40.2)	100 (39.1)	60 (42.3)	0.244	54 (45.8)	49 (41.5)	0.88
	T2a	69 (17.3)	50 (19.5)	19 (13.4)		17 (14.4)	16 (13.6)	
	T2b	104 (26.1)	60 (23.4)	44 (31.0)		31 (26.3)	36 (30.5)	
	T3	43 (10.8)	30 (11.7)	13 (9.2)		13 (11.0)	12 (10.2)	
	T4	22 (5.5)	16 (6.2)	6 (4.2)		3 (2.5)	5 (4.2)	
N stage (*n*, %)	N0	291 (73.1)	183 (71.5)	108 (76.1)	0.386	96 (81.4)	88 (74.6)	0.272
	N1	107 (26.9)	73 (28.5)	34 (23.9)		22 (18.6)	30 (25.4)	
M stage (*n*, %)	M0	309 (77.6)	205 (80.1)	104 (73.2)	0.149	88 (74.6)	85 (72.0)	0.769
	M1	89 (22.4)	51 (19.9)	38 (26.8)		30 (25.4)	33 (28.0)	
Surgery (*n*, %)	No	194 (48.7)	111 (43.4)	83 (58.5)	0.005	67 (56.8)	63 (53.4)	0.695
	Yes	204 (51.3)	145 (56.6)	59 (41.5)		51 (43.2)	55 (46.6)	
LND* (*n*, %)	N13	59 (14.8)	47 (18.4)	12 (8.5)	0.001	10 (8.5)	12 (10.2)	0.607
	N4	42 (10.6)	34 (13.3)	8 (5.6)		5 (4.2)	8 (6.8)	
	No	297 (74.6)	175 (68.4)	122 (85.9)		103 (87.3)	98 (83.1)	
Radiation (*n*, %)	No	326 (81.9)	210 (82.0)	116 (81.7)	1	100 (84.7)	97 (82.2)	0.726
	Yes	72 (18.1)	46 (18.0)	26 (18.3)		18 (15.3)	21 (17.8)	
Chemotherapy (*n*, %)	No	196 (49.2)	121 (47.3)	75 (52.8)	0.339	68 (57.6)	63 (53.4)	0.6
	Yes	202 (50.8)	135 (52.7)	67 (47.2)		50 (42.4)	55 (46.6)	
Marital status (*n*, %)	Married	248 (62.3)	167 (65.2)	81 (57.0)	0.06	71 (60.2)	66 (55.9)	0.779
	Unknown	18 (4.5)	14 (5.5)	4 (2.8)		3 (2.5)	4 (3.4)	
	Unmarried	132 (33.2)	75 (29.3)	57 (40.1)		44 (37.3)	48 (40.7)	
Age (year) (*n*, %)	<70	253 (63.6)	152 (59.4)	101 (71.1)	0.026	71 (60.2)	82 (69.5)	0.173
	≥70	145 (36.4)	104 (40.6)	41 (28.9)		47 (39.8)	36 (30.5)	
Size (cm), (*n*, %)	<4.6	212 (53.3)	135 (52.7)	77 (54.2)	0.857	61 (51.7)	62 (52.5)	1
	≥4.6	186 (46.7)	121 (47.3)	65 (45.8)		57 (48.3)	56 (47.5)	

*Note*: “N1–3″ indicated the patients underwent surgery and the number of LND in the range of 1–3, “N4^+^” indicated the patients underwent surgery and the number of LND in the range of >4; PSM: propensity score matching.

Abbreviations: AJCC, the American Joint Commission on Cancer; *LND. Lymph node dissection; IPTW: inverse probability of treatment weighting; “N0” indicated the patients did not undergo surgery or underwent surgery with no LND.

The results of comparison by log‐rank test suggested a beneficial trend in the noncirrhotic group compared with the cirrhotic group with regard to overall survival (OS) time (23 (20–29) versus 13 (11–17) *p* = 0.023, Figure 2A) and cancer‐specific survival (CSS) (26 (22–35) vs 15 (12–20) *p* = 0.0041, Figure 2D). In addition, this finding was also confirmed by performing analysis between the two groups in terms of OS (17 months [13–26] vs 12 months [10–20] *p* = 0.52, Figure 2B) and CSS (22 months [15–30] vs 14 months [11–23] *p* = 0.15, Figure 2E) in the PSM population and in IPTW pseudo‐population (OS: 20 months [15–26] vs 13 months [11–17] *p* = 0.163, Figure 2C and CSS: 22 months [17–30] vs 15 months [12–22] *p* = 0.059, Figure 2F).

**FIGURE 3:**
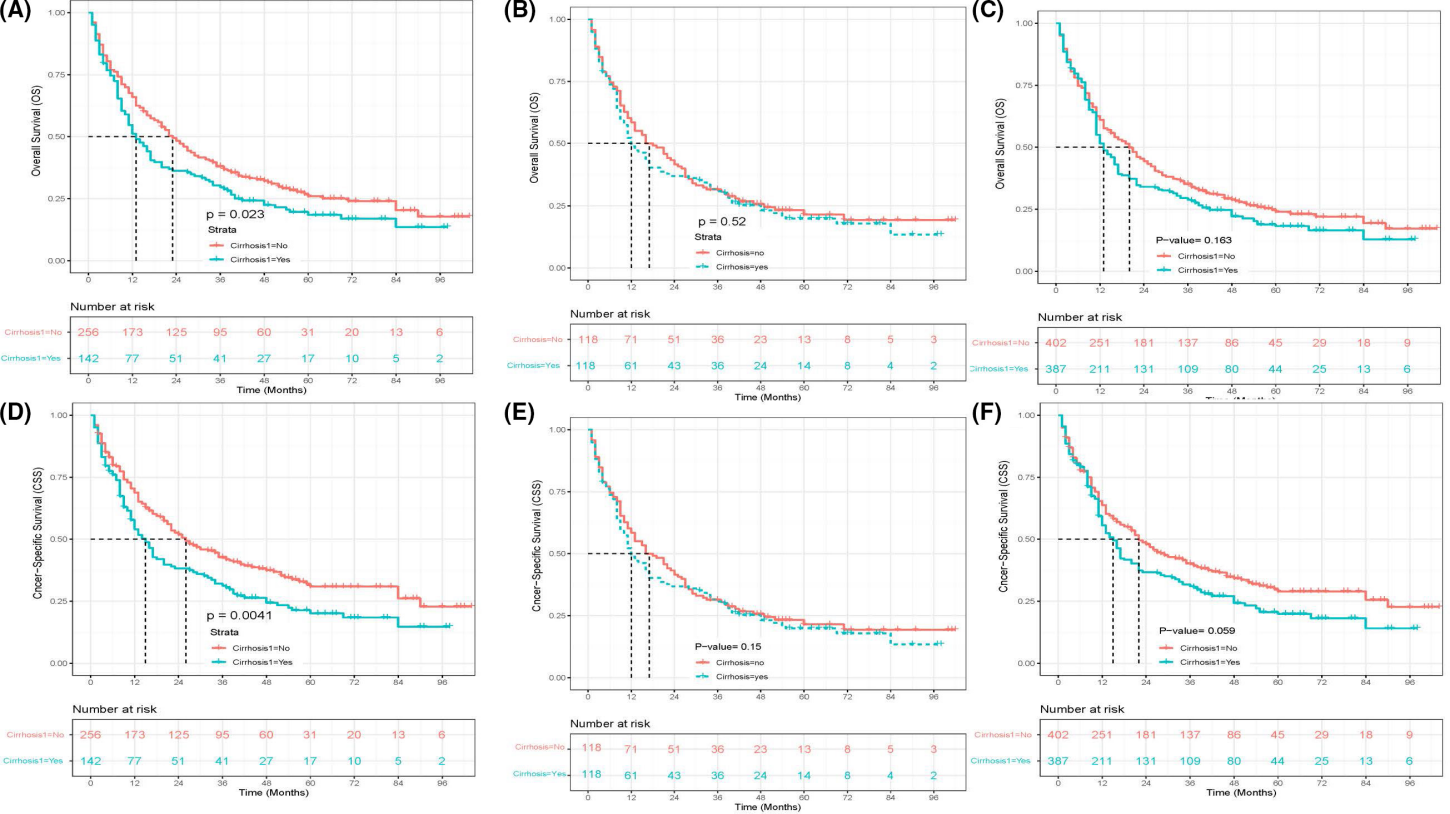
Overall survival (OS) in the primary cohort, PSM cohort, and IPTW pseudo‐cohort (A, B, C), cancer‐specific survival (CSS) in the primary cohort, PSM cohort, as well as IPTW pseudo‐cohort (D, E, F). PSM, propensity score matching; IPTW, inverse probability of treatment weighting

Additionally, before PSM, the 1‐, 3‐, and 5‐year OS rates in the noncirrhotic group and the cirrhotic group were 66.0%, 38.0%, and 26.0%, respectively, and 51.2%, 29.6%, and 18.5%. The 1‐, 3‐, and 5‐year CSS rate in the noncirrhotic cohort was 68.8%, 42.7%, and 30.9%, respectively, versus 54.0%, 31.3%, and 20.1% in the cirrhotic cohort. Following PSM, the 1‐, 3‐, and 5‐year overall survival (OS) rates were 58.5%, 31.4%, and 21.5%, respectively, in the noncirrhotic group and 48.8%, 30.7%, and 19.9%, respectively, in the cirrhotic cohort group, whereas the 1‐, 3‐, and 5‐year CSS rates in the noncirrhotic cohort were 62.2%, 37.9%, and 31.4%, respectively, than 52.2%, 32.9%, and 22.0% in the cirrhotic cohort. Taken together, it seemed that ICC patients in the cirrhotic group may have better OS and CSS than those in the noncirrhotic group (Figure 3).

After PSM, there were 118 matched pairs of patients included for further analysis. Of these patients who did not experience surgery for ICC, cirrhosis was not associated with the prognosis of the patients with ICC. However, this is in contrast with the results observed in the subgroup of ICC patients underwent surgical resection, it was noted that the noncirrhotic patients showed a more beneficial trend in OS and CSS than that of the cirrhotic patients.

On the other hand, the result of multivariate Cox regression analysis demonstrated that the cirrhosis showed no statistical significance on OS (hazard ratio [HR] 1.10; 95% confidence interval [CI] 0.84–1.43) and CSS (HR 1.240; 95% CI 0.9–1.71), while AJCC TNM staging, surgery, and chemotherapy were significant prognostic factors for OS and CSS (Tables 2 and 3).

**TABLE 2:** Univariate and multivariate Cox regression analyses on overall survival (OS) in the PSM‐matched population

Characteristics	Levels	Univariate analysis			Multivariate analysis		
		HR	95% CI	*p* value	HR	95% CI	*p* value
Sex	Female	Ref					
	Male	0.83	0.62–1.12	0.232			
Race	Black	Ref			Ref		
	Other	1.61	0.69–3.77	0.268	1.15	0.58–2.3	0.692
	White	1.92	0.9–4.11	0.09	1.54	0.86–2.76	0.149
Grade	III/IV	Ref			Ref		
	I/II	0.6	0.4–0.9	0.013	0.65	0.45–0.95	0.026
	Unknown	1.26	0.88–1.8	0.2	0.88	0.6–1.3	0.523
T stage	T1	Ref			Ref		
	T2a	2.32	1.5–3.59	<0.001	1.97	1.38–2.81	<0.001
	T2b	3	2.1–4.28	<0.001	1.92	1.34–2.74	<0.001
	T3	3.96	2.44–6.42	<0.001	2.42	1.49–3.94	<0.001
	T4	1.56	0.63–3.87	0.342	2.85	1.5–5.43	0.001
N stage	N0	Ref			Ref		
	N1	3.07	2.18–4.33	<0.001	1.25	0.88–1.79	0.214
M stage	M0	Ref			Ref		
	M1	5.02	3.6–7.01	<0.001	2.37	1.62–3.46	<0.001
Surgery	No	Ref			Ref		
	Yes	0.19	0.14–0.27	<0.001	0.28	0.18–0.43	<0.001
LND*	N13	Ref			Ref		
	N4	1.2	0.47–3.06	0.7	1.34	0.71–2.54	0.372
	N0	2.54	1.41–4.57	0.002	1.28	0.79–2.09	0.319
Radiation	No	Ref					
	Yes	1.57	1.08–2.27	0.018	1.05	0.79–1.39	0.736
Chemotherapy	No	Ref					
	Yes	1.35	1.01–1.81	0.043	0.54	0.4–0.72	<0.001
Cirrhosis	No	Ref			Ref		
	Yes	1.1	0.83–1.47	0.51	1.1	0.84–1.43	0.502
Size (cm)	<4.6	Ref			Ref		
	≥4.6	2.37	1.77–3.19	<0.001	1.29	0.97–1.73	0.084
Age (years)	<70	Ref					
	≥70	1.26	0.94–1.7	0.122			
Marital status	Married	Ref					
	Unknown	1.53	0.67–3.5	0.311			
	Unmarried	1.27	0.95–1.71	0.11			

Abbreviations: CI confidence intervals; HR hazard ratios; Ref, reference.

**TABLE 3:** Table Univariate and multivariate Cox regression analyses on cancer‐specific survival (CSS) in the PSM‐matched population

Characteristics	Levels	Univariate analysis			Multivariate analysis		
		HR	95% CI	*p* value	HR	95% CI	*p* value
Sex	Female	Ref					
	Male	0.85	0.62–1.17	0.33			
Race	Black	Ref			Ref		
	Other	2.06	0.78–5.47	0.146	2.32	0.8–6.72	0.121
	White	2.38	0.97–5.8	0.058	3.31	1.25–8.77	0.016
Grade	III/IV	Ref			Ref		
	I/II	0.56	0.37–0.85	0.007	0.63	0.39–1.03	0.065
	Unknown	1.16	0.8–1.69	0.429	0.84	0.54–1.32	0.454
T stage	T1	Ref			Ref		
	T2a	2.13	1.31–3.45	0.002	1.86	1.1–3.14	0.02
	T2b	3.09	2.11–4.51	<0.001	1.92	1.27–2.92	0.002
	T3	4.3	2.61–7.09	<0.001	2.34	1.33–4.1	0.003
	T4	1.81	0.72–4.52	0.206	1.92	0.67–5.49	0.224
N stage	N0	Ref			Ref		
	N1	3.54	2.48–5.04	<0.001	1.5	0.95–2.36	0.081
M stage	M0	Ref			Ref		
	M1	5.6	3.94–7.94	<0.001	2.23	1.38–3.61	0.001
Surgery	No	Ref			Ref		
	Yes	0.2	0.14–0.28	<0.001	0.24	0.14–0.4	0.001
LND*	N13	Ref			Ref		
	N4^+^	1.23	0.45–3.38	0.693	1.43	0.49–4.15	0.515
	N0	2.67	1.4–5.08	0.003	1.65	0.78–3.48	0.19
Radiation		Ref			Ref		
	Yes	1.49	1–2.23	0.049	1.05	0.67–1.63	0.843
Chemotherapy		Ref			Ref		
	Yes	1.46	1.07–1.99	0.017	0.5	0.33–0.75	0.001
Cirrhosis	No	Ref			Ref		
	Yes	1.26	0.92–1.71	0.149	1.24	0.9–1.71	0.192
Size(cm)	<4.6	Ref			Ref		
	≥4.6	2.55	1.86–3.5	<0.001	1.38	0.97–1.95	0.074
Age (years)	<70	Ref			Ref		
	≥70	1.17	0.85–1.62	0.323	0.73	0.51–1.06	0.098
Marital status	Married	Ref			Ref		
	Unknown	1.44	0.58–3.55	0.431	1.09	0.42–2.86	0.86
	Unmarried	1.34	0.98–1.83	0.071	1.39	0.99–1.95	0.061

## 4 DISCUSSION

Generally, hepatocellular carcinoma (HCC) mainly results from cirrhosis caused by HBV, HCV, intrahepatic bile duct stones, etc.^5^ In contrast, the underlying mechanism of ICC is still elusive, and a proportion of patients develop cirrhosis, which is thought to be an important risk factor for ICC, while there are usually noncirrhotic factors such as liver flukes, congenital biliary anomalies, metabolic syndrome, and biliary tract surgeries identified in most patients with ICC.^4^, ^5^, ^6^ Whether there is a distinct difference in oncological results in ICC patients with cirrhosis and without cirrhosis remains obscure. Therefore, the knowledge of the correlation between cirrhosis and ICC needs to be further broadened and deepened, and the current information of the study should be considered in future therapeutic decisions.

**FIGURE 4:**
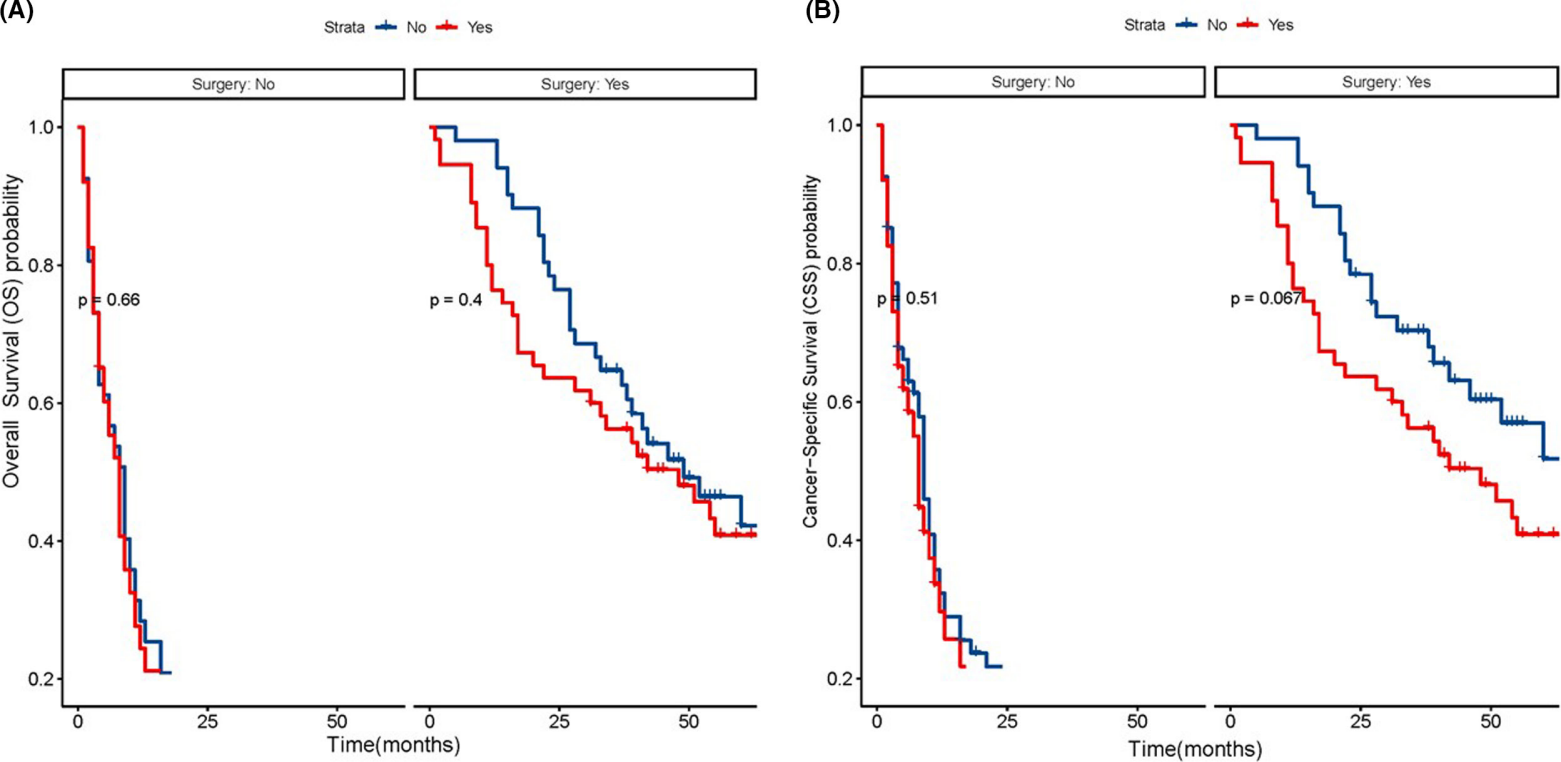
OS (A) and CSS (B) between the cirrhotic and noncirrhotic patients in surgical and nonsurgical groups with ICC following PSM

To date, studies regarding the results of ICC have provided contradictory outcomes, particularly in patients with and without cirrhosis, reflecting huge intercenter variability. There was no difference in survival between the noncirrhotic group and the group with compensated cirrhosis in the study conducted by Daniel and colleagues, which was in line with Francesco and colleagues' previous study, while Li et al.^18^, ^19^ found that cirrhosis was an independent factor for poor prognosis in patients with ICC who underwent surgical resection. We applied the statistical method of PSM to reduce the risk of a possible selection bias based on the patients with ICC extracted from the SEER database to explore whether cirrhosis influences the long‐term prognosis of patients with ICC. Consequently, there was no significant difference in median OS between the two groups, which indicated that cirrhosis itself may not result in a worse long‐term prognosis for patients with ICC. Additionally, cirrhosis may have a prognostic difference in ICC patients who have undergone and did not undergo surgery. We classified the patients into subgroups of surgical and nonsurgical groups to explore whether cirrhosis could pose a distinct difference in the long‐term oncological outcomes of ICC patients who underwent surgical intervention. As a result, we found that cirrhosis did not impose an adverse impact on the OS and CSS of nonsurgical patients with ICC (Figure 4A,B). This was in contrast with the result observed in ICC patients who underwent surgery. Thus, the above‐mentioned finding was in line with the result of the study conducted by Zhan and colleagues, whose study was also based on the population of the SEER database.^13^

Furthermore, there is a difference in the short‐term outcome of ICC patients between the cirrhotic and noncirrhotic groups. Before PSM, it is noteworthy that the ICC patients with no cirrhosis had a higher opportunity for surgical resection and a more advanced AJCC TNM staging than those in the cirrhotic group, which suggests that cirrhosis may limit the choice of treatment for the ICC patients in this study. Generally, patients with cirrhosis have a poorer hepatic function and are prone to have higher short‐term postoperative mortality and morbidity than noncirrhotic patients.^7^, ^8^, ^9^ Existing studies have indicated that cirrhotic liver results in a higher risk of hepatectomy liver failure due to insufficient functional liver remnant remains for maintaining postoperatively normal physiological functions.^9^, ^10^, ^11^ Radical surgical resection is believed to be the only potentially curative management for patients with ICC, while a lower chance of receiving radical resection means worse oncological outcomes. Furthermore, cirrhotic patients with ICC may have a lower likelihood of receiving lymphadenectomy and anatomical resection, which are believed to correlate with the favorable oncological prognosis of ICC.^15^, ^23^ Given the absence of information on liver function tests and postoperative complications in the SEER database, we cannot further examine the short‐term impact of cirrhosis on patients with ICC who underwent surgical resection in this study. Taken together, cirrhotic patients with ICC may have contributed to a poorer short‐term outcome than ICC patients without cirrhosis.

Currently, the topic of whether lymphadenectomy (LND) should be performed for ICC remains controversial. Despite accumulating evidence supporting a therapeutic benefit of routine LND in hepatic resection for ICC, other studies have demonstrated that LND did not influence the prognosis of patients with ICC who underwent surgery.^24^, ^25^, ^26^ In addition, several previous studies have also demonstrated that tumor size is an important prognostic factor for ICC.^27^, ^28^ The multivariable Cox regression analysis of our study showed that surgery, AJCC TNM stage, and race of white were independent risk factors associated with OS of ICC instead of LND or tumor size.

There are several shortcomings in this study. First, this is a retrospective study with a natural bias of data, despite the PSM being used to reduce potential bias. Second, some clinicopathological factors commonly cited that may affect prognosis (such as surgical margins, microvascular invasion [MVI], and carbohydrate antigen 19‐9 [CA19‐9]) were not included in this study due to the absence of relevant records in the SEER database, so multivariable analysis was performed adjusted for available clinical variables.^29^, ^30^ Similarly, although several histopathological details have been demonstrated to be vital predictors of survival, we cannot carry them into the Cox proportional hazard analysis because histological subtypes of ICC are not offered in the public‐use SEER data set. Data on cancer recurrence, and Child–Pugh class was not provided in the public‐use SEER data set.

## 5 CONCLUSION

Collectively, it seems that the noncirrhotic patients with ICC have similar relative overall survival (OS) but better cancer‐specific survival (CSS) than that of the cirrhotic patients.

## ETHICS STATEMENT

Data (anonymized) were collected from the SEER database, and no consent was required for this study.

## CONFLICT OF INTEREST

All authors report no conflicts of interest in this work.

## AUTHOR CONTRIBUTIONS

J.W. and Y.Y. contributed to the idea of the study. The manuscript was mainly written by J.W., M.Z., and Y.Q. S.S., B.Z. and W.W. revised the manuscript with constructive suggestions.

## DISCLOSURE

The authors declare that no financial relationships exist.

## Data Availability

The data sets generated and/or analyzed during the current study are available in the SEER registry through the SEER*Stat software (version 8.3.9, http://seer.cancer.gov/seerstat/).
